# *Daphnia magna* and *Gammarus pulex,* novel promising agents for biomedical and agricultural applications

**DOI:** 10.1038/s41598-022-17790-z

**Published:** 2022-08-11

**Authors:** Abdelrahman M. Khattab, Hamdy A. Abo-Taleb, Amer M. Abdelaziz, Mohamed A. M. El-Tabakh, Mohamed M. M. El-feky, Mohammed Abu-Elghait

**Affiliations:** 1 Botany and Microbiology Department, Faculty of Science, Al-Azhar University, Nasr City, Cairo, 11884 Egypt; 2 Zoology Department, Faculty of Science, Al-Azhar University, Nasr City, Cairo, 11884 Egypt; 3 Aquatic Resources, Natural Resources Studies and Research Department, College of High Asian Studies, Zagazig University, Zagazig, 44519 Egypt

**Keywords:** Biotechnology, Microbiology, Zoology

## Abstract

Various studies have shown the importance of using different types of Zooplankton biomasses as an additional substance in the diet of fish. In addition, the drainage water of the fish cultures could be used in plant irrigation. In this study, biomasses of water flea *Daphnia magna and Gammarus pulex* collected and tested, for the first time, their effect against pathogenic microorganisms and on plant germination. The results showed significant antibacterial activity of *D. magna* and *G. pulex* against *Staphylococcus aureus* and *Pseudomonas aeruginosa* bacteria, as well as antifungal activity against *Alternaria solani* and *Penicillium expansum*, which gives the possibility to be used as biocontrol against these bacteria and plant pathogenic fungi. Furthermore, both animals showed positive activity in the germination rate of *Vicia faba* seed, reaching 83.0 ± 3.5 and 86.0 ± 3.8%, respectively. In conclusion, the biomasses of *D. magna* and *G. pulex* are promising and effective agents for their use in the medical field against some pathogenic microbes and as stimulators of plant growth.

## Introduction

Zooplankton plays a critical role in aquaculture, influencing survival of livestock under harsh environmental conditions and growth depending on various conditions^1^. In addition, zooplankton contributes to the aquatic food chain as an intermediator, feeding upon bacteria, fungi, and algae, while turning fed by numerous invertebrates, birds, and fishes^2^. Zooplankton are used as an environmental indicator for water quality, pollution, and the eutrophication situation^3^. Recently, plankton biotechnology has gained extensive significance, because of its rich constituents involved in active primary and secondary metabolites^4^. The current applied studies on zooplankton communities mainly focused on their composition as a rich source of pigments, vitamins, essential fatty acids, proteins, carbohydrates, and biologically active primary and secondary metabolites^5^. Many studies mentioned the importance of using *Daphnia magna (*Straus, 1820) and *Gammarus pulex* (Linnaeus, 1758) as an alternative substance for protein in the fish diet at different fish farms^2,6–12^.

The water flea *D. magna* is a micro-crustacean zooplankton widespread in freshwater bodies. Recently, it was used for feeding fish fry in aquaculture as it has a high nutritional composition that varies according to the culture medium and viability degree of phytoplankton^2,11–14^. The crustacean amphipod *G. pulex* is widespread worldwide^11^ and one of the most common freshwater macroinvertebrates. *G. pulex* has a central role in organic matter degradation^15,16^, and risk assessment^17^, besides its contribution to the food net^18^. It contains high levels of protein, fats, and amino acids^19^, thus *Gammarus* meal enhances the feed intake, immune response, stress resistance, and growth performance in fish^20^. *Gammarus* is used as a cheap alternative substrate for animal protein in the diet of highest value fish^21^. Therefore, it is important to explore the effect of *D. magna* and *G. pulex* biomass against the most common pathogenic microbes that could infect either the fed-fish or humans as the end-consumer. In addition, it is important to study their effect on plant germination, because the drainage water of fish farms is loaded with biomass and is recycled for irrigating plants.

Pathogenic microbes and infectious diseases have become one of the major problems in the medical field, leading to the death of many people worldwide^22^. Antibiotics resistance is counted as one of the most parameters affecting human health, particularly after the emergence of multi-drug-resistant (MDR) pathogens^23,24^. The wrong usage of antibiotics and the deficiency of capabilities and scientific tools to improve such drugs had led scientists to explore alternative natural molecules with potential antimicrobial activity^25^.

Several types of bacteria and fungi can enter, individually or together, vital matrix called biofilm through a microbial-molecular communication system that regulates the virulence traits of these microbes and introducing infections by biofilm-forming microbes via medical devices^26^. The biofilm is a complex network of lipids and proteins used to protect microbes against a broad range of antibiotics and the host immune system^27–29^. Thus, it is necessary to search for new treatment substrates such as natural molecules to provide a new vision in treatment and limit the spread of MDR pathogens^28,30–32^.

Different species of fungi cause the major common plant diseases, mostly controlled by synthetic fungicides^33^. Whereas repeated fungicide leads to the rapid evolution of fungal resistance against the fungicide, creating a high demand for exploring a natural alternative molecule^34^.

Broad beans or faba beans (*Vicia faba*) are considered one of the most important winter crops with high nutritional value, especially in Egypt^35^. The main fungal diseases infecting *Vicia faba* are wilting, root rot, leaf spot, and even plant death caused by species of diverse fungal genera such as *Alternaria, Fusarium, Ascochyta, Colletotrichum, Rhizopus, Pythium, Rhizoctonia,* and *Clonostachys*^36,37^. Hence, finding an eco-friendly substrate will be helpful in biocontrol plant diseases.

The discovery of natural antimicrobials might meet the consumer demand for environmentally friendly materials avoiding chemicals with harmful side effects^38,39^. There has been a promising trend for scientific research and industrial applications of biotechnology and marine compounds^40^. In this context, this study was conducted to studying for the first time the antibacterial, antifungal, and antibiofilm activities of the dried biomasses of *G. pulex,* and *D. magna* to be used safely in the fish diet and medical field, and limit the spread of some pathogenic bacterial and fungal microbes in addition to investigating the effect of these biomasses on plant seed germination. This report will open the door for further applications and valorization for other types of zooplankton as an inexpensive and safe alternative substrate.

## Materials and methods

### Tested animals

*Daphnia magna* and *Gammarus pulex* were collected from northern Egypt, Lake Mariout (31° 7.5′ N, 29° 47.1′ E) in June 2019; the salinity in this part of the lake is rather stable around 8‰^39^.

Cladoceran species, *Daphnia magna,* and Amphipods, Gammarus *spp*., were collected with a standard plankton net of 200 µm mesh size, which was lowered vertically to a shallow bottom then pulled up to the water surface^5,7^. Then the net content was placed into plastic containers filled with lake water and identify *D. magna* and *G. pulex* through dissecting microscope. The samples were identified by traditional morphological and biochemical methods. The identified *D. magna* and *G. pulex* species were kept in 1001 transplant plastic holding containers that had dechlorinated tap water, with a salinity of 8% as in nature, for 24 h acclimatization before starting the cultivation experiments. After cultivation for a certain period under standard conditions, according to the methods described by Abo-Taleb et al.^11^ both species were dried at 70 °C for 48 h to be ready for further experiments^2^.

### Chemical analyses of the tested animals

The chemical composition of *D. magna* and *G. pulex* including moisture, carbohydrate, crude protein, fibers, crude fat, calories, and ash, was analyzed according to the standard methods of AOAC (1990)^6^**.** The moisture content was estimated by drying the samples to constant weight at 70 °C in a drying oven for 48 h. Whereas the nitrogen content was measured using an Automatic Kjeldahl system (UDK 139, VELP Scientifica). However, the lipid content was determined by ether extraction in multiunit extraction Soxhlet apparatus. The ash was determined by combusting dry samples in a muffle furnace at 550 °C for 3 h. Vitamins A, B2, B6, B12, D, E, and folic acid, besides the antioxidants such as tannic acid and β-carotene, have been determined in the cultivated *D. magna* and *G. pulex* to evaluate their nutritional value^5^.

### Biological Activities of D. magna and G. pulex

#### Antibacterial and MIC determination

Antibacterial activities of *D. magna* and *G. pulex* were evaluated using an agar well diffusion assay on Muller-Hinton agar plates according to CLSI guidelines against human pathogenic bacterial strains *Pseudomonas aeruginosa* ATCC 27,853 and *Escherichia coli* ATCC8739, which represented Gram-negative bacteria, whereas *Bacillus subtilis* ATCC 6051 and *Staphylococcus aureus* ATCC 6538 were used to represent Gram-positive bacteria. The inhibition zone diameter was specified in millimeter (mm)^41^**.** The Micro-dilution method in micro-titer plates (MTP) was applied to determine the minimum inhibitory concentration (MIC) of active *D. magna* and *G. pulex* against tested pathogenic bacteria. Accordingly, *D. magna* and *G. pulex* were diluted in 1% DMSO at various concentrations (0.009–5.0 mg mL^−1^, *w*/*v*) and tested against the pathogenic bacterial strains. Briefly, 1:100 (*v*/*v*) overnight cultures of the test strains were added to 200 µl of Mueller–Hinton broth media dispersed in MTP wells with/without *D. magna* and *G. pulex.* Then, the plates were incubated with shaking at 120 rpm at 37 °C for 24 h. The lowest concentration of *D. magna* and *G. pulex* which inhibited bacterial growth was considered the MIC^42,43^. All experiments were performed in triplicates.

#### Antibiofilm assay

To analyze the anti-biofilm activity of the two animal species, a Microtiter plate (MTP) assay was used. *D. magna* and *G. pulex* were tested against two known biofilm-producing strains: *Pseudomonas aeruginosa* ATCC 27,853 and *Staphylococcus aureus* ATCC 6538. Sub-inhibitory concentrations (0.01–1.0 mg mL^−1^) of *D. magna* and *G. pulex* were loaded in a flat bottom MTP, containing Tryptic-Soy broth media (TSB) supplemented with 1% glucose and mixed well. The tested strains were cultured and incubated overnight in TSB at 37 °C and after incubation diluted to reach the turbidity of 1.5 × 10^8^ CFU mL^−1^ then inoculated into MTP wells containing different concentrations of the tested biomasses and incubated at 37 °C for 48 h^44^. After the incubation period, the growth density was measured at OD 620 nm. Subsequently, the floated cells were transferred without troubling the formed biofilm and the plates were washed three times with sterilized phosphate-buffered saline (PBS) pH 7.4 to remove excess residue of floated unbounded cells. The biofilms in all wells were fixed with 200 µl of methanol 95% for 10 min. Then, crystal violet (CV 0.3% *w*/*v*) was added to each well using a multi-channel micropipette (CAPP, Germany), and the plates were incubated for 15 min at room temperature. After that, the excess CV was removed, and the wells were gently washed with sterile distilled water. For the quantitative detection of biofilm formation, the adherent biofilm-bounded CV was assayed by eluting in 30% acetic acid and measured at OD 540 nm using an automated microplate reader (Tecan, Elx800-USA). The treated wells were compared with that of the untreated control. the untreated well inoculated with the test organism is considered the positive control while the negative control is the uninoculated media. All control and experiment groups were performed in triplicates.

#### The activity against plant pathogenic fungi

The antifungal activity of *D. magna* and *G. pulex* was performed by the agar well method^45^ against plant pathogenic fungi strains *Fusarium oxysporum* RCMB (008 001 “2”), *Penicillium expansum* RCMB (001 001 “1”), *Alternaria solani* RCMB (009 003), *Aspergillus niger* RCMB (002 007 “2”), *Aspergillus fumigatus* RCMB (002 008 “2”), and *Rhizoctonia solani* RCMB (031 001). Briefly, 10 mg mL^−1^ of *D. magna* or *G. pulex* crude powder were placed separately into the wells of the plates. The plates were incubated for 5 days at 30 °C. Antifungal activity was evaluated by measuring the diameter of the inhibition zone for each strain. Each strain was tested in triplicates.

#### The seed germinations test

The *faba* bean cv. Giza 716 was obtained from the Legume Research Department, Field Crop Institute, Agricultural Research Center, Egypt. The seeds of *Vicia faba* cv. Giza 716 identification was confirmed by the Botanical herbarium, Botany and Microbiology Department, Faculty of Science Al-Azhar University. The collection of plant material complies with the institutional, national, and international guidelines and legislation. equal size were surface sterilized by submersion in 10% sodium hypochlorite solution for 10 min, soaked in distilled water at 25 °C for 24 h, and then allowed to germinate in Petri dishes. One piece of filter paper and 5 mL of different concentrations (1, 0.5, 0.25, 0.125, 0.0625, and 0.031 mg mL^−1^) of *D. magna* and *G. pulex* were placed in distilled water in other Petri dishes. The germinating seeds were transferred to filter paper with 10 seeds per dish and a minimum distance of 1 cm between each seed (three replicates for each treatment). Distilled water was used for the control (untreated) experiments. For the germination rate and root growth investigation, the seeds were allowed to germinate for one week, then the seed germination percentage was calculated, and the seedling root length was measured. Four replicates were carried out for each treatment. Treated and control seeds were evaluated for record the percentage of seed germination with the following formula^46^:

$$Germination \left(\%\right)= \frac{The\,\, number\,\, of\,\, germinated\,\, seeds }{The \,\,total\,\, number \,\,of\,\, used\,\, seeds}\times 100$$

### Statistical analysis

Three replicates were performed for each assay, and the standard error was calculated. all resulting values were the averages of three independent replicates. The differences between a sample and the corresponding control were analyzed by using Student’s *t*-test and the differences were considered significant if the *p* values were ≤ 0.05.

### Ethical approval

This article does not contain any studies with human participants performed by any of the authors.

## Results and discussions

### Chemical analyses of tested animals

The chemical content of the two tested animals was analyzed to determine their prospective efficiency for industrial applications. Biochemical composition of *D. magna* and *G. pulex* is given in Table 1 based on three independent readings. The results point to higher chemical content of *D. magna* in protein, fibers, nitrogen-free extract, and both types of energy, while *G. pulex* had higher chemical content in fats, ash, and carbohydrate (Table 1). The chemical composition of *Gammarus* can be affected by several factors such as age, season, habitats, region, and drying process after harvesting^21^. Based on their high content of protein and other ingredients, *D. magna and G. pulex* were the proper alternatives for fish meal in feeding fish larvae^47^.

**Table 1:** Biochemical composition of *D. magna* and *G. pulex* biomasses.

Ingredients *(% of their dry weight)*	*Daphnia magna*	*Gammarus pulex*
Moisture	6.8	6.1
Crude protein (CP, %)	45.1	41.8
Fat or Ether extract (EE, %)	11.4	13.6
Crude fiber (CF, %)	3.3	2.4
Ash (%)	13.4	19.6
Nitrogen-free extract (NFE, %)	26.8	22.6
Total carbohydrate (%)	22.1	25.4
Gross energy (GE, Kcal kg^−1^)	4829.45	4646.90
Digestible energy (DE, Kcal kg^−1^)	3622.09	3485.18
NFE = 100—(CP + CF + EE + Ash%); according to Jobling^44^ DE = GE × 0.75		

### Vitamins and antioxidant content

*D. magna* and *G. pulex* contain vitamins and antioxidants required to improve the health status of fish larvae. The vitamins contained in 100 g of *D. magna* were Vitamin A (279.53 IU), B2 (218.44 mg), B6 (120.84 mg), B12 (222.61 mg), D (22.59 mg), E (98.12 mg), and folic acid (139.08 µg). The antioxidant found in 100 g of *D. magna* were β-carotene (6,811.90 IU) and tannic acid (137.65 mg).

**Figure 1:**
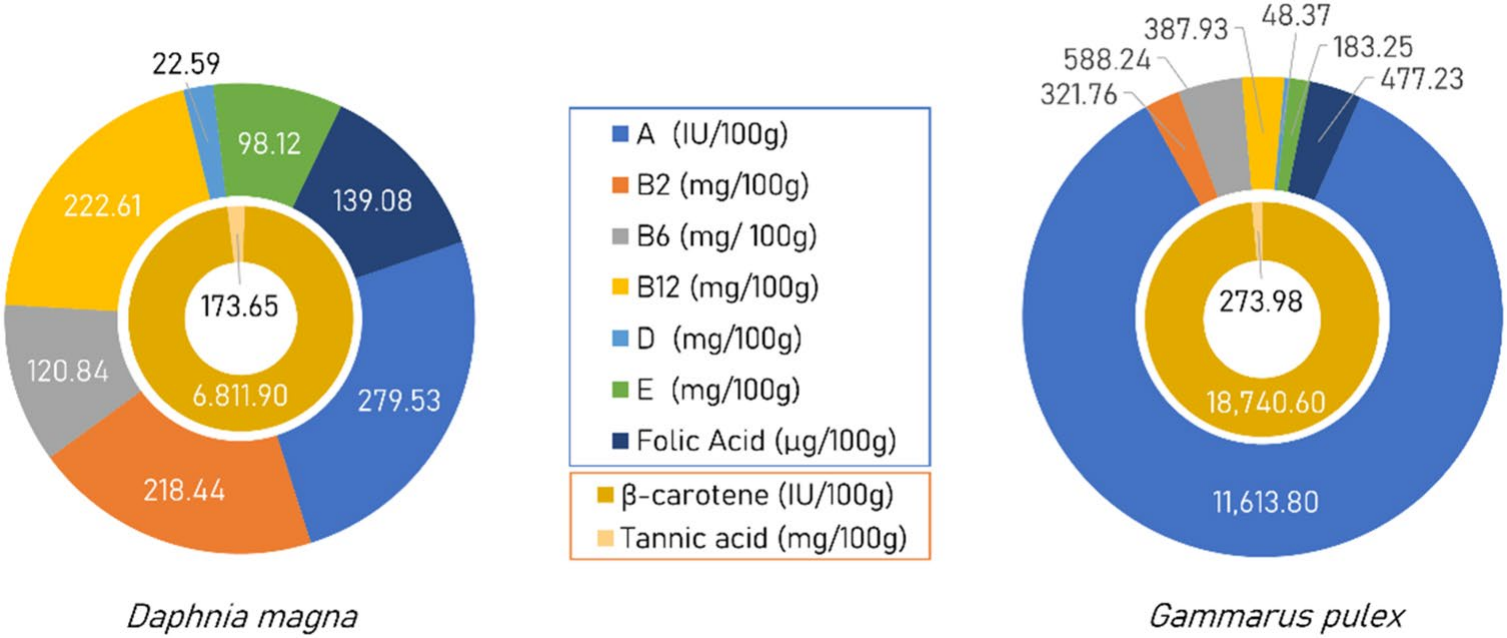
Vitamin and antioxidant content of *D. magna* and *G. pulex.*

The vitamins contained in 100 g of *G. pulex* were: Vitamin A (11 IU), B2 (613.80 mg), B6 (321.76 mg), B12 (288.24 mg), D (48.37 mg), E (183.25 µg), and folic acid (477.23 µg). The antioxidant found in 100 g of *G. pulex* were β-carotene (18,740.60 IU) and tannic acid (273.98 mg) (Fig. 1).

Therefore, the major constituents of *D. magna* and *G. pulex* biomasses were vitamin-A and β-carotene. The vitamin content in *D. magna* obtained from this study was lower than that mentioned by El-Feky et al.^2^, although the antioxidant contents were higher. On the other hand, the vitamin and antioxidant contents of *G. pulex* of this study showed a lower variation than mentioned by Abo-Taleb et al*.*^11^.

### Antibacterial activity and MIC determination

The inhibitory action of *D. magna* and *G. pulex* biomasses are indicated by the diameter of the inhibition zone formed (Table 2). The results indicated that *G. pulex* has better inhibitory effects against Gram-positive and Gram-negative pathogenic bacteria than *D. magna.* The inhibitory action for *D. magna* and *G. pulex* biomasses may be due to the reaction of the biomasses with bacterial protein by combining the thiol (-SH) group leading to the inactivation of proteins and bacterial growth. Therefore, it is necessary to test the MIC of *D. magna* and *G. pulex* for each bacterial strain (Table 2). bioagents with lowest MIC values is candidate to be effective antimicrobial agents^48^. Results showed that the MIC for *D. magna* was 2.0 mg mL^−1^ against both *P. aeruginosa* and *S. aureus.* Moreover, MIC for *G. pulex* was 2.0 mg mL^−1^ against *P. aeruginosa* and *S. aureus*, respectively. Hence, biomasses have the same activity against Gram positive and negative bacteria*.* However, the mechanism of bacterial growth reduction through the interaction of *D. magna* and *G. pulex* still needs further studies.

**Table 2:** Antibacterial activity of *D. magna* and *G. pulex* presented as the inhibition zone and Minimal inhibition concentration (MIC).

Tested microorganisms	*D. magna*		*G. pulex*	
	Inhibition zone (mm)	MIC (mg mL^−1^)	Inhibition zone (mm)	MIC (mg mL^−1^)
*Escherichia coli*	0	NA	0	NA
*Pseudomonas aeruginosa*	13.25 ± 0.51	2.0	16.52 ± 0.33	2.0
*Bacillus subtitles*	0	NA	0	NA
*Staphylococcus aureus*	11.45 ± 0.32	2.0	12.22 ± 0.40	2.0

NA: No activity. ±: Standard deviation, Gram negative bacteria represented by: *Escherichia coli* and *Pseudomonas aeruginosa,* Gram positive bacteria represented by: *Bacillus subtitles* and *Staphylococcus aureus*.

### The antibiofilm activity

**Figure 2:**
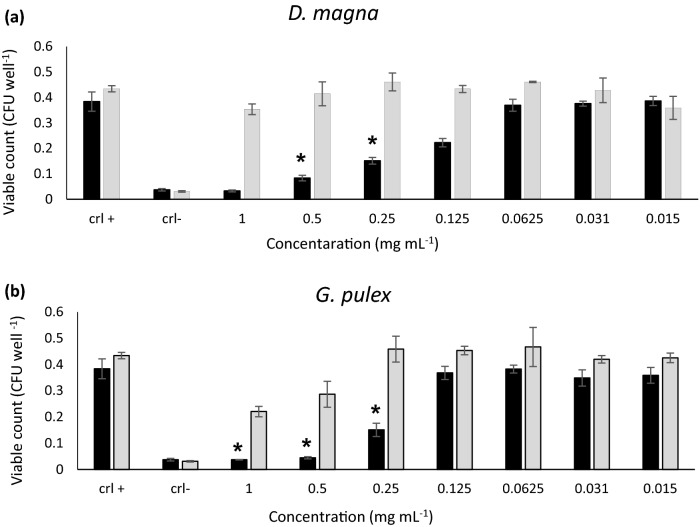
Biofilm inhibition activity of *D. magna* (**a**) and *G. pulex* (**b**) samples, against *S. aureus* and *P. aeruginosa.* Significant differences are indicated by * (P < 0.05). crl + : positive control. crl-: negative control.

In this work, the antibiofilm activity of the tested biomass exhibited varied effects against different bacterial strains (Fig. 2). Accordingly, *G. pulex* and *D. magna* biomass affected the biofilm formation of *Pseudomonas aeruginosa* at concentrations lower than the inhibitory concentration, while the biomass didn't exhibit any antibiofilm activity against *S. aureus.* MIC values of the *D. magna* and *G. pulex* against *P. aeruginosa* and *S. aureus* were 2.0 mg mL^−1^. The sub-lethal concentrations of *D. magna* and *G. pulex* exhibited antibiofilm activity against *P. aeruginosa* with significant differences (p < 0.05) in a dose-dependent manner where the concentrations 1, 0.5, 0.25, and 0.125 mg mL^−1^ of *D. magna* were reducing the biofilm formation of *P. aeruginosa* with 91.43, 78.31, 60.63, and 42.04%, respectively, while the same concentrations didn't affect the biofilm formation of *S. aureus* (Fig. 2a). On the other hand, the concentrations of 1, 0.5, and 0.25 mg mL^−1^ of *G. pulex* reduced the biofilm formation of *P. aeruginosa* by 90.38, 88.41, and 60.71% respectively, whereas the concentrations 1 and 0.5 mg mL^−1^ only affect the biofilm formation of *S. aureus* by 49.1 and 33.9% (Fig. 2b). The ability of these biomasses to reduce the biofilm formation of this opportunistic pathogens in a dose-dependent manner at sub-inhibitory concentrations motivating the researcher for more exploration to investigate the active compounds in these biomasses that may interfere with the biofilm formation pathway or the virulence regulation system in bacteria such as quorum sensing system. Our findings make these compounds promising molecules that could use as biofilm preventive agents. Interestingly, the primary stage of biofilm inhibition was observed at the MIC values^41^. The previous study explained the antibiofilm activity which showed deformation, external cell roughness, and cell wall shrinkage of bacterial cells^49^.

### The activity against plant pathogenic fungi

This study investigated the activity of *D. magna* and *G. pulex* biomasses against six plant pathogenic fungi (*A. solani, A. niger, A. fumigatus, F. oxysporum, P. expansum,* and *R. solani*). The results showed that *D. magna* and *G. pulex* had antifungal activity against two plant pathogenic fungi (*A. solani* and *P. expansum)* at 10 mg mL^−1^ (Table 3). The inhibition zone diameters formed by the effect of *D. magna* biomass were 10.31 ± 0.88 and 10.41 ± 0.51 mm for *A. solani* and *P. expansum*, respectively, while *G. pulex* exhibited antifungal activity with inhibition zone diameters of 10.88 ± 0.72 and 9.61 ± 0.43 mm for the same fungal strains *A. solani* and *P. expansum*, respectively. The biomasses may inhibit the fungal growth by affecting cellular functions, causing distortion in fungal hyphae, or preventing the expansion of conidia and conidiophores, consequently leading to cell death^50^. These different responses of tested plant pathogenic fungi may be due to the resistance of some strains and the sensitivity of others^51^ . The sensitivity of fungal plant pathogens may be altered by the sensitive target site by mutant or changes in amino acids^52^. The activity of *D. magna* and *G. pulex* biomasses against Alternaria *solani* and *Penicillium expansum* which cause severe damage to plants may contribute to the production of a safe bio fungicide in the future to protect the plant from this fungus.

**Table 3:** Antimicrobial activities of *D. magna* and *G. pulex* against plant fungal pathogens.

Tested microorganisms	Inhibition zone (mm)	
	*D. magna*	*G. pulex*
*Alternaria solani RCMB 009 003*	10.31 ± 0.88	10.88 ± 0.72
*Aspergillus niger RCMB 002 007 “2”*	0	0
*Aspergillus fumigatus RCMB 002 008 “2”*	0	0
*Fusarium oxysporum RCMB 008 001 “2”*	0	0
*Penicillium expansum RCMB 001 001 “1”*	10.41 ± 0.51	9.61 ± 0.43
*Rhizoctonia solani RCMB 031,001*	0	0

### Seed germinations test

**Figure 3:**
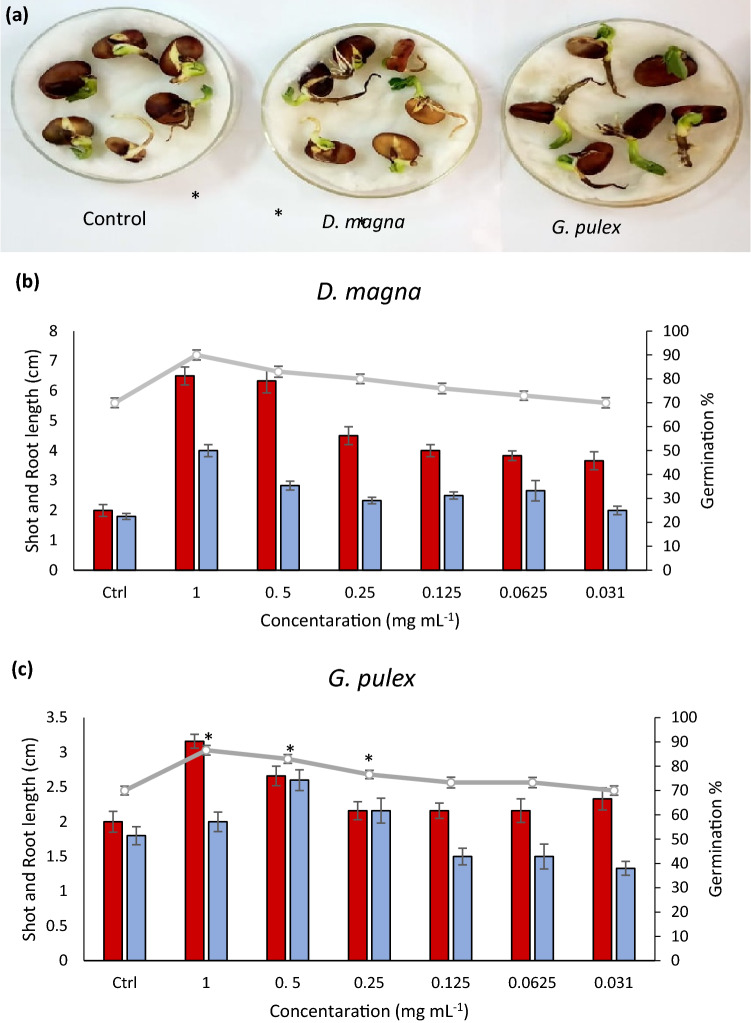
Effect of different concentrations of *D. magna* and *G. pulex* on *faba* bean (*V. faba*) seed germination rate and shoot and root length. (**a**) Seeds in the petri dish with control, *D. magna,* and *G. pulex*. (**b**) The effect of different concentrations of *D. magna.* (**c**) The effect of different concentrations of *G. pulex.* Significant differences are indicated by * (P < 0.01).

The experiment showed a positive effect with *G. pulex* and *D. magna* on seed germination presence of *V. faba*. *D. magna* and *G. pulex* increased the germination percentage with significant differences (P < 0.01) when compared with the control at 1, 0.5, and 0.25 mg mL^−1^ **(**Fig. 3). *D. magna* enhanced the germination rate by 90, 83, and 80%, respectively (Fig. 3b), while with *G. pulex* the increase in germination rate was 86.6, 83, and 76.6%, respectively (Fig. 3c). Furthermore, *D. magna* showed high enhancement of root length with the highest effect recorded at 1 mg mL^−1^ of 222%, while *G. pulex* increased root length by 111% compared to control. On the other hand, the 0.031 mg mL^−1^ concentration did not show any positive effect on seed germination by either *D. magna* or *G. pulex*. These results relate to several studies which reported that seaweed extracts improved the *V. faba* morphological characters such as shoot and root lengths. The use of water flea, *D. magna*, and the crustacean amphipod, *G. pulex*, as complementary protein and lipid sources in the transitional diet for Common Carp (*Cyprinus carpio* L.) nurseries^53^ affect the growth and yield of faba bean (*V. faba* L.) positively. These components are involved in the primary metabolism of building and maintaining plant cells and viability, resulting in growth enhancement. The more inductive effect of *D. magna* and *G. pulex* on *Vicia faba* vegetative growth parameters including shoot height, was explained by the presence of folic acid in *D. magna* and *G. pulex*. For more, *D. magna* and *G. pulex* used as nutraceuticals through enhancement biochemical mechanisms and production of secondary metabolites^54^. The previous study carried by Cauchie, et al.,^55^. *al.,* Chitosan plays an effective role in the enhancement of plant growth and inductive defense factors in many plants through regulation of the most important metabolic processes as Carbon and Nitrogen Metabolism^56^. Another reason for the induction effect of Chitosan contents of *D. magna* and *G. pulex* on *Vicia faba* vegetative growth is that Chitosan reduced water uptake by minimizing plant transpiration. Moreover, nutrients, mineral vitamins, and antioxidants resulted in the regulation of *Vicia faba* metabolic processes, especially against stress conditions^57,58^.

‏

## Conclusion

In this study, isolated biomass of *D. magna* and *G. pulex* showed obvious ability to inhibit several pathogens involving Gram-positive and Gram-negative bacteria, as well as inhibition against some plant pathogenic fungi. The current report considers the first biotechnological study of the current micro and macroinvertebrates. The biomass from *D. magna* and *G. pulex* exhibited the possibility of applying them in the medical and agricultural fields limiting the spread of some pathogenic microbes and enhancing plant germination.

## Data Availability

All authors declare that the data supporting the findings of this study are available within the article.
